# Soybean (*Glycine max* L.) triacylglycerol lipase GmSDP1 regulates the quality and quantity of seed oil

**DOI:** 10.1038/s41598-019-45331-8

**Published:** 2019-06-20

**Authors:** Masatake Kanai, Tetsuya Yamada, Makoto Hayashi, Shoji Mano, Mikio Nishimura

**Affiliations:** 1 Laboratory of Organelle Regulation, National Institute for Basic Biology, Okazaki, 444-8585 Japan; 2 Graduate School of Agriculture, Hokkaido University, Sapporo, 060-8589 Japan; 3 Department of Bioscience, Nagahama Institute of Bio-Science and Technology, Nagahama, 526-0829 Japan; 4 Department of Basic Biology, SOKENDAI (The Graduate University for Advanced Studies), Okazaki, 444-8585 Japan; 5 Department of Biology, Faculty of Science and Engineering, Konan University, Kobe, 658-8501 Japan

**Keywords:** Plant biotechnology, Plant physiology

## Abstract

Seeds of soybean (*Glycine max* L.) are a major source of plant-derived oils. In the past, improvements have been made in the quantity and quality of seed oil. Triacylglycerols (TAGs) are the principal components of soybean seed oil, and understanding the metabolic regulation of TAGs in soybean seeds is essential. Here, we identified four soybean genes encoding TAG lipases, designated as *SUGAR DEPENDENT1-1* (*GmSDP1-1*), *GmSDP1-*2, *GmSDP1-3* and *GmSDP1-4*; these are homologous to *Arabidopsis thaliana SDP1* (*AtSDP1*). To characterize the function of these genes during grain filling, transgenic lines of soybean were generated via RNA interference to knockdown the expression of all four *GmSDP1* genes. The seed oil content of the transgenic soybean lines was significantly increased compared with the wild type (WT). Additionally, fatty acid profiles of the WT and transgenic soybean lines were altered; the content of linoleic acid, a major fatty acid in soybean seeds, was significantly reduced, whereas that of oleic acid was increased in transgenic soybean seeds compared with the WT. Substrate specificity experiments showed that TAG lipase preferentially cleaved oleic acid than linoleic acid in the oil body membrane in WT soybean. This study demonstrates that the GmSDP1 proteins regulate both the TAG content and fatty acid composition of soybean seeds during grain filling. These results provide a novel strategy for improving both the quantity and quality of soybean seed oil.

## Introduction

Plant oils have diverse uses; they have been utilised as dietary oils, soaps, and industrial materials. In addition, recent explosion of population and extensive interest in biofuels has accelerated the demand for plant oils^1,2^. Because the total agricultural area of the world remains unchanged (FAOSTAT: http://faostat3.fao.org/home/), improvement in plant oil yield per unit area is necessary to address the increase in demand. Engineering crops with high oil content is required worldwide. Oilseed crops, such as soybean (*Glycine max* L.), rape (*Brassica napus*), peanut (*Arachis hypogaea*) and sesame (*Sesamum indicum*), are cultivated for the production of plant oils. Because plant oils, in most cases, are extracted from seeds, it is therefore important to enhance the oil content of seeds.

Triacylglycerols (TAGs) are one of the primary components of seed oils, and their biosynthetic pathway has been extensively studied in seeds^3–7^. In plants, fatty acids are synthesised in plastids, and subsequently TAGs are synthesised from fatty acids and glycerol in the endoplasmic reticulum (ER). The synthesised TAGs are then stored in oil bodies, which are storage organelles for oil. Recent advances in biochemical and molecular biological studies have revealed the TAG biosynthetic pathway in seeds^8,9^. Several reports show that modifications of the TAG biosynthetic pathway significantly increase oil production in seeds^10–13^, indicating that the modification of this pathway is key to improving oil production in plants. The TAG degradation pathway has also been shown to affect the oil content of seeds. TAG degradation is essential for the post-germinative growth of oilseed crop plants^14–16^. The first step in TAG degradation is the hydrolysis of TAGs, which is catalysed by SUGAR DEPENDENT1 (SDP1), a TAG lipase present in oil body membranes^17^. TAG hydrolysis produces fatty acids, which are imported into the peroxisome by a peroxisomal ABC transporter PED3/CTS/PXA1^18–20^ and converted into energy for post-germinative growth^14,16,21,22^. The *sdp1* mutants have been shown to exhibit defective TAG degradation post-germination^17,23^. The TAG degradation pathway is activated not only post-germination but also during seed development. Defective SDP1 has been shown to enhance the seed oil content in Arabidopsis (*Arabidopsis thaliana*)^24^, rapeseed^25^, and Jatropha (*Jatropha curcas*)^26^. These reports suggest that TAG turnover is highly active in developing seeds, and the suppression of TAG degradation enhances the oil content of seeds.

In addition to the quantity of TAGs, the quality of TAGs is important. TAG quality is highly dependent on its fatty acid composition, which differs widely in plant species^2^. Olive oil and rapeseed oil, which are used as dietary oils, contain a large portion of oleic acid (18:1), whereas cottonseed oil and soybean oil contain a large amount of linoleic acid (18:2). By contrast, the primary fatty acid in palm kernel oil, which is used in soaps, is lauric acid (12:0). The fatty acid composition of seed oil determines its usage and value; therefore, conducting research aimed at modifying the fatty acid composition of seed oil is encouraged^2,4,7^. In fact, several crops with altered fatty acid composition such as canola, a rapeseed variant with low erucic acid (22:1) content, are wildly cultivated. This indicates that the modification of fatty acid composition is necessary for the establishment of valuable oilseed crops.

Soybeans (*Glycine max)* are one of the most important crops for vegetable protein supplies, and their breeding for increased seed protein has been studied. At the same time, soybeans are also a major oilseed crop, and soybean oil is the world’s second mostly highly produced oil after palm oil (FAOSTAT: http://faostat3.fao.org/home/). However, the oil content of soybean seed is relatively low compared with that of other oilseed crops. Therefore, studies focussed on identifying ways to increase the seed oil content in soybean are required^27^. Polyunsaturated fatty acids (PUFAs), including linoleic and linolenic acids, are susceptible to oxidation. Large amounts of linoleic and linolenic acids reduce the oxidative stability of soybean oil; thus, it is important to reduce the amount of these fatty acids^27^. Further investigation of the TAG metabolic pathway is needed to develop soybean cultivars with high oil and low PUFA content.

In this study, we identified *SDP1* genes in soybean and generated transgenic soybean lines with suppressed *SDP1* expression, specifically during seed development. Our data show that GmSDP1 regulates both the oil content and fatty acid composition of soybean seeds.

## Results

### Identification and characterisation of *SDP1* genes in soybean

**Figure 1:**
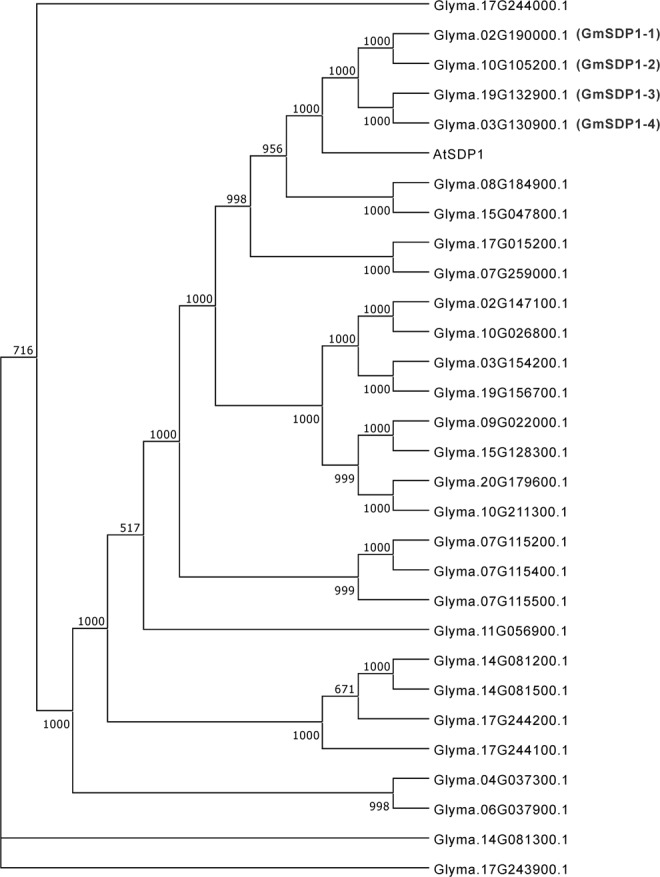
Phylogenetic analysis of the triacylglycerol (TAG) lipase in *Arabidopsis thaliana* (AtSDP1) and 29 lipases containing patatin-like phospholipase domain in soybean. Amino acid sequences of all proteins were obtained from Phytozome v12.1.6. Phylogenetic analysis was performed using MEGA7. Bootstrap values were determined based on 1,000 repetitions.

This study aimed to understand TAG degradation during seed development in soybean. The reference genome of soybean cv. Williams 82 is 1.1 Gbp, and approximately 75% of the genes exist as multiple copies^28^. To identify soybean genes orthologous to Arabidopsis *SDP1* (*AtSDP1*) gene, amino acid sequences containing patatin-like phospholipase domain (PF01734), were acquired from the soybean genome using Phytozome v12.1.6^29^. Phylogenetic analysis of 29 sequences containing the domain of PF01734 from soybean genome was performed, which revealed that four lipase sequences (Glyma.02g190000.1, Glyma10g105200.1, Glyma19g132900.1 and Glyma03g130900.1) belonged to the same clade as AtSDP1 (Fig. 1); we designated these four sequences as GmSDP1-1, GmSDP1-2, GmSDP1-3 and GmSDP1-4, respectively. All four GmSDP1 amino acid sequences showed over 70% homology with the AtSDP1 sequence (Supplementary Fig. S1).

**Figure 2:**
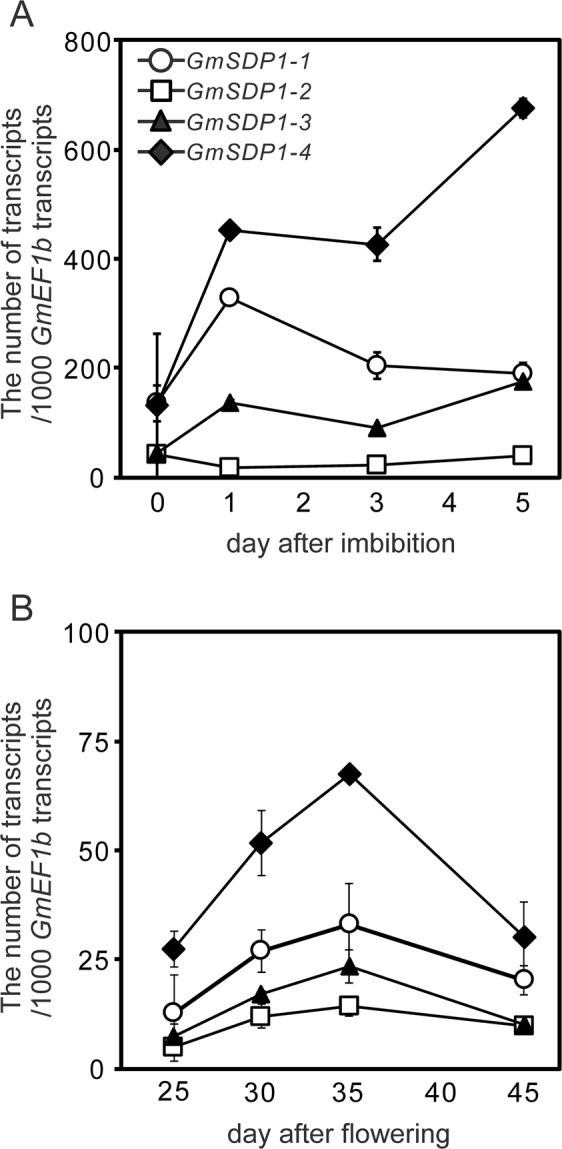
Quantitative analysis of *GmSDP1-1, GmSDP1-2, GmSDP1-3* and *GmSDP1-4* expression levels during seed germination and development. Expression levels of *GmSDP1-1, 1-2, 1-3* and *1-4* were measured via quantitative real-time PCR (qRT-PCR) analysis during germination (**A**) and seed development (**B**). The *GmEF1b* gene was used as an internal control^41^. Standard curves were generated with plasmids containing *GmSDP1-1, 1-2, 1-3, 1-4* or *GmEF1b* cDNA. Values represent mean ± standard deviation (SD) of three independent experiments.

The expression of *GmSDP1* genes was analysed during seed germination. After 1 day of seed imbibition, expression of *GmSDP1-4*, *GmSDP1-1* and *GmSDP1-3* increased by 4-, 3- and 2-fold, respectively, compared with those at 0 day of seed imbibition (Fig. 2A). After 3 days of seed imbibition, expression of *GmSDP1-3* and *GmSDP1-4* was similar to those at day 1, whereas the expression of *GmSDP1-1* was significantly lower than that at day one (Fig. 2A). After 5 days of seed imbibition, expression of *GmSDP1-3* and *GmSDP1-4* was higher than those at day 3 (Fig. 2A), whereas the expression of *GmSDP1-1* was similar to that at day 3 (Fig. 2A). No increase in *GmSDP1-2* expression was detected during germination (Fig. 2A). Expressions of the *GmSDP1* genes were also measured during seed development. In developing seeds, expressions of all *GmSDP1* genes increased until 35 days after flowering (DAF) but decreased at 45 DAF (Fig. 2B). These results show that multiple copies of *GmSDP1* are transcribed in soybean seeds.

### Establishment of transgenic soybean lines with suppressed *GmSDP1* gene expression

**Figure 3:**
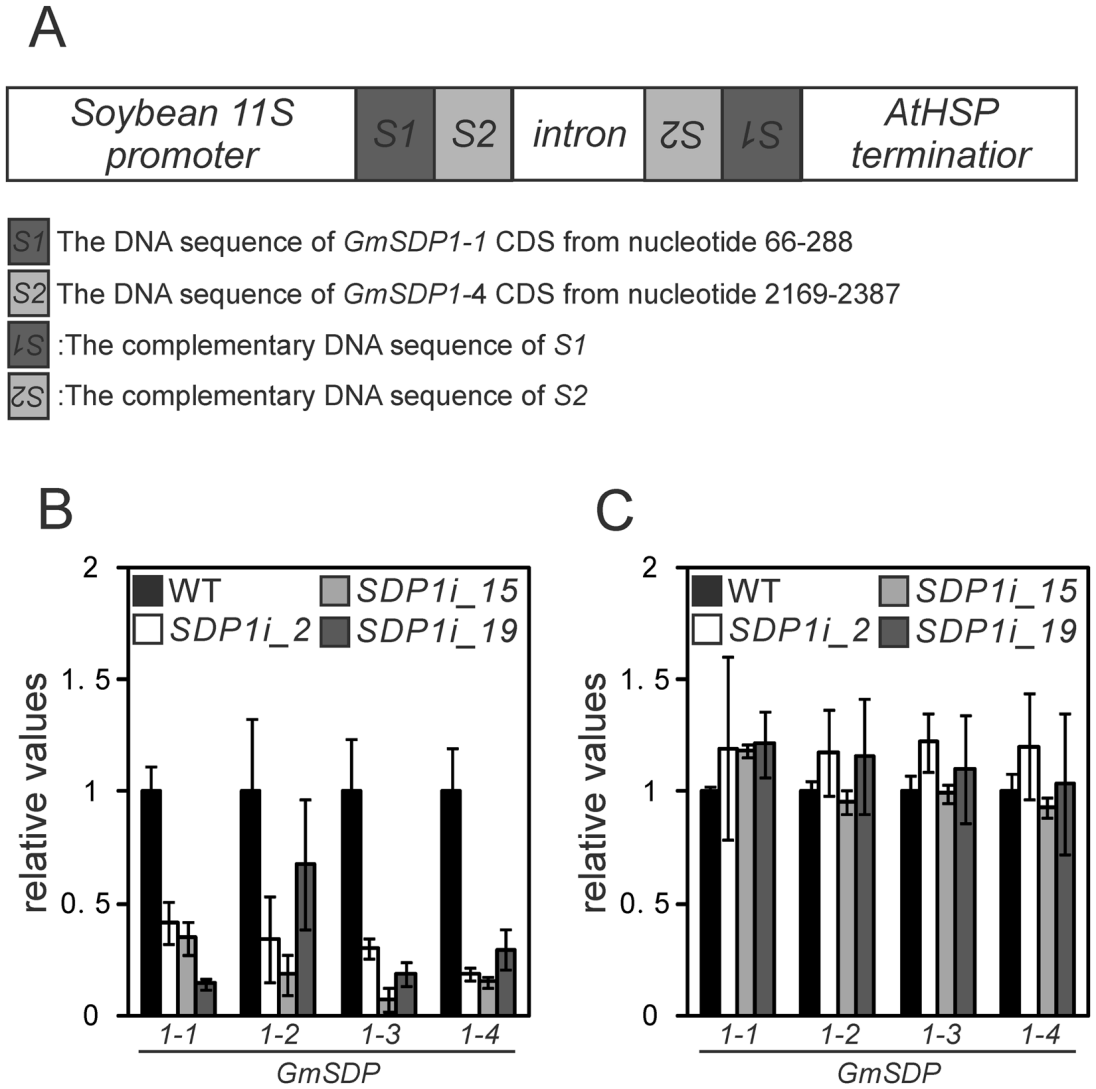
Establishment and characterisation of *GmSDP1* knockdown lines in soybean. (**A**) Schematic diagrams of the RNAi construct used for *GmSDP1* knockdown. (**B**,**C**) qRT-PCR analysis of relative expression levels of *GmSDP1-1, 1-2, 1-3* and *1-4* in seeds at 35 DAF (**B**) and 3-day-old seedlings (**C**) of the WT and transgenic lines. *GmEF1b* was used as an internal control. Standard curves were generated using plasmids containing *GmSDP1-1, 1-2, 1-3, 1-4* or *GmEF1b* cDNA. Values represent mean ± SD of three independent experiments.

Multiple copies of *GmSDP1* were expressed during seed development. To verify the function of *GmSDP1* genes during seed development, we generated transgenic soybean lines via RNA interference (RNAi) to knockdown the expression of all four *GmSDP1* genes. The patatin-like phospholipase domain (PF01734) is present in all 29 lipases (Fig. 1); therefore, the DNA sequence of this domain was an unsuitable candidate for RNAi-mediated knockdown of the *GmSDP1* genes. We, therefore, screened highly conserved sequences specifically present in the *GmSDP1* genes. Two such sequences were identified at the 5′ and 3′ termini of these genes (Supplementary Fig. S2). The sequence identified at the 5′ terminus (*S1*; Fig. 3A) was cloned from the coding sequence (CDS) of *GmSDP1-1*, and the sequence at 3′ terminus (*S2*; Fig. 3A) was cloned from *GmSDP1-4* CDS. The cloned *S1* and *S2* sequences were fused in tandem, and the fused sequence was cloned into an RNAi vector under the control of soybean *11S globulin* gene promoter, one of the genes encoding a major seed storage protein in soybean (Fig. 3A). The RNAi vector was introduced into WT soybean plants via infiltration using *Agrobacterium tumefaciens*, and three independent transgenic lines were established (*SDP1i*_*2*, _15 and _19). The germination percentage of the transgenic lines was approximately 80%, which was similar to that of the WT (data not shown). Expressions of each *GmSDP1* gene were measured at 35 DAF, and significant reduction in the expression of each gene was observed in the transgenic lines during seed development (Fig. 3B). On the other hand, expressions of all four *GmSDP1* genes in transgenic lines were similar to those in the WT during germination (Fig. 3C). These results show that the expression of *GmSDP1* genes in transgenic lines was significantly suppressed, specifically during seed development.

### Seed yield and oil content of transgenic soybean lines

**Figure 4:**
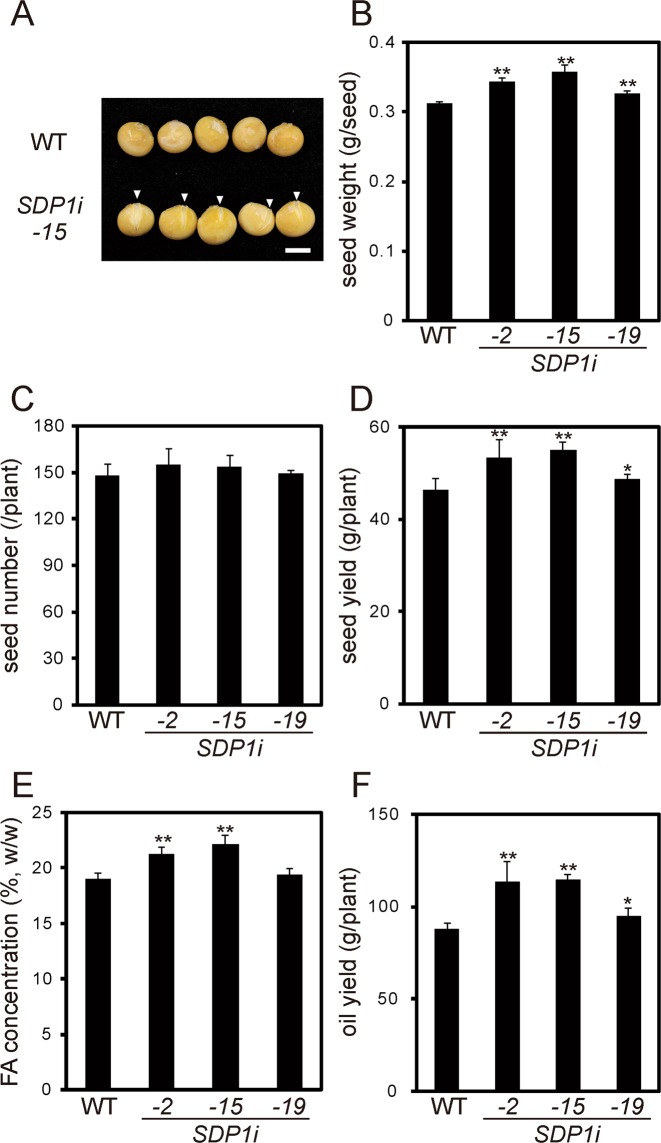
Productivity of *GmSDP1* knockdown lines. (**A**) Images of WT and transgenic *SDP1i-15* seeds. Scale bars = 0.5 mm. **(B**) Weight of transgenic seeds. Values represent mean ± SD of three independent experiments with 130–150 seeds harvested from individual plants. Significant difference between WT and transgenic lines was determined using Student’s *t*-test and is denoted as **(*P* < 0.05). **(C)** Seed numbers harvested from transgenic lines. Values represent mean ± SD of three individual plants. **(D)** Seed yield of transgenic lines. Values represent mean ± SD of three individual plants. Significant difference between WT and transgenic lines was determined using Student’s *t*-test and is denoted as *(*P* < 0.1) or **(*P < *0.05). **(E)** Fatty acid (FA) concentrations in seeds of transgenic lines measured via gas chromatography–mass spectrometry (GC–MS). Values represent mean ± SD of three independent experiments, with 20 seeds per experiment. Significant difference between WT and transgenic plants determined using Student’s *t*-test is denoted as **(*P* < 0.05). **(F)** Oil yield of transgenic lines estimated from the seed yield and FA concentration. Values represent mean ± SD of three individual plants. Significant difference between WT and transgenic lines determined using Student’s *t*-test is denoted as *(*P* < 0.1) or **(*P* < 0.05).

Transgenic lines of soybean were grown in the growth chamber, and seed production was investigated. Seeds of line *SDP1i*_15 were larger than that of WT, and seed coat rupture was also observed (arrow heads in Fig. 4A). The weight of WT seeds was 0.313 g, whereas seeds of lines *SDP1i*_2, *_15* and *_19* weighed 0.343, 0.357 and 0.326 g, respectively (Fig. 4B). By contrast, seed number showed no differences between the WT and transgenic lines (Fig. 4C). The seed yield, which represents weight of seeds harvested from a plant, of transgenic lines was significantly higher than that of the WT; seed yield of lines *SDP1i*_2, _15, and _19 was 15.2%, 18.6% and 5.2%, respectively, higher than that of the WT (Fig. 4D). Additionally, the seed oil content of the transgenic lines *SDP1i_*2, *_*15 and*_*19 was 2.2%, 3.1% and 0.4% higher than that of the WT, respectively (Fig. 4E). We also estimated the oil yield per plant, based on the seed yield and oil content. The oil yield of lines *SDP1i_2*, *_15* and*_19* was 29.2%, 30.5% and 7.8% higher, respectively, than that of the WT (Fig. 4F). Taken together, these results show that the knockdown of multiple *GmSDP1* genes increases the seed oil content in soybean.

### Fatty acid profiles of transgenic soybean lines

**Figure 5:**
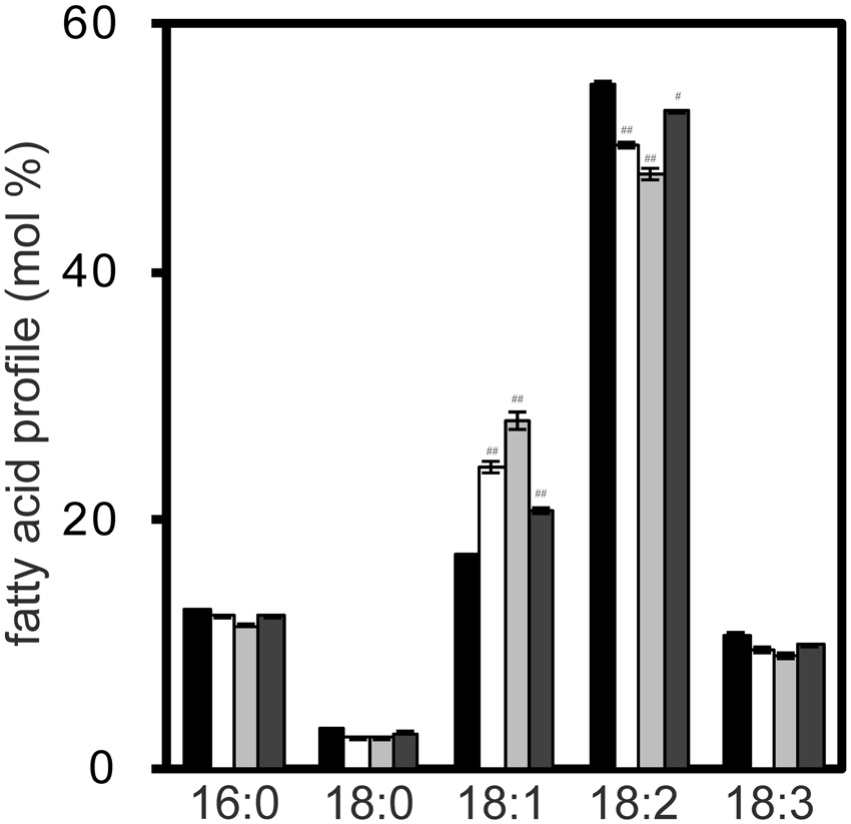
Fatty acid profiles of *GmSDP1* knockdown lines. Fatty acid profiles of TAGs extracted from mature seeds. TAGs were extracted from seeds using thin layer chromatography (TLC). Fatty acid profiles of TAGs were measured via GC–MS. Values represent mean ± SD of three independent experiments, with 20 seeds per experiment. Significant difference between WT and transgenic plants determined using Student’s *t*-test is denoted as ^##^(*P < *0.01) or ^###^(*P* < 0.001).

In addition to the seed oil content, the fatty acid composition is also important for the breeding of superior oilseed crops. We thus analysed the fatty acid composition of seed TAGs. Results showed that linoleic acid (18:2) was a major component of TAGs, comprising 56% of the total fatty acids in WT seeds; however, it was significantly reduced by 4.9%, 7.3% and 2.1% in the transgenic lines *SDP1i*_*2*, _*15*, and _*19*, respectively, compared with the WT (Fig. 5). By contrast, the content of oleic acid (18:1) was significantly increased by 6.9%, 10.6% and 3.4% in the transgenic lines *SDP1i_2*, *_15*, and *_19*, respectively, compared with the WT (Fig. 5). No major differences in the amount of palmitic acid (16:0), stearic acid (18:0) and linolenic acid (18:3) were detected between the WT and transgenic lines (Fig. 5). These results indicate that *GmSDP1* genes regulate the ratio of oleic acid to linoleic acid in TAGs.

**Figure 6:**
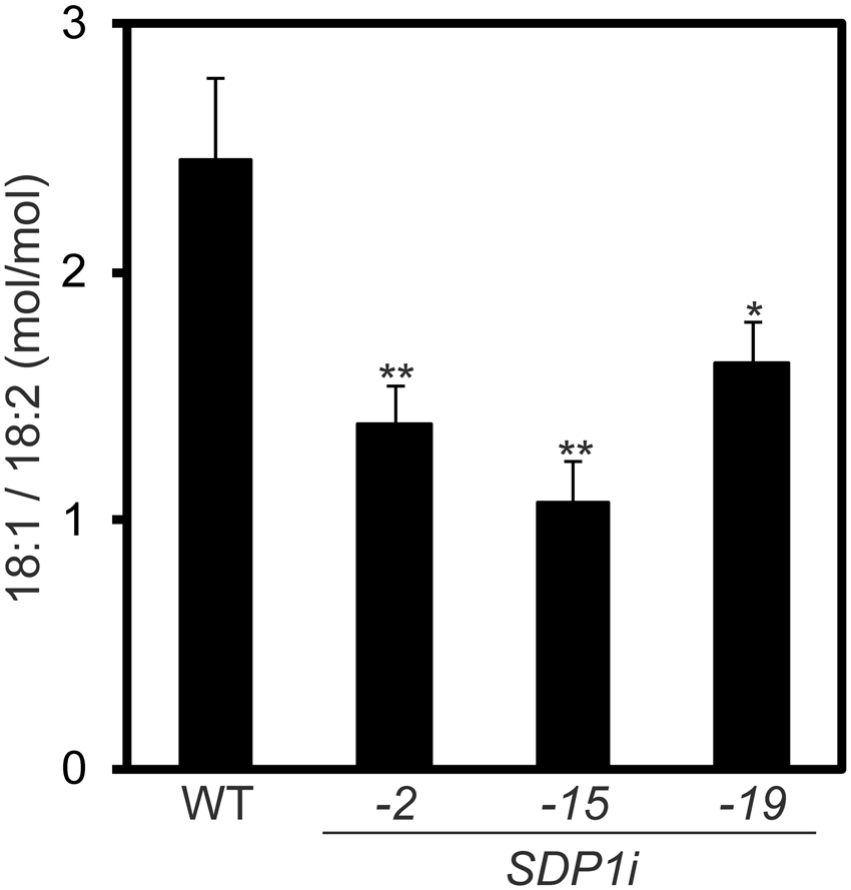
Substrate specificity of TAG lipase in oil body membranes in soybean. Substrate preference of TAG lipase was determined using oil body membranes isolated from seeds at 35 DAF. The isolated membranes were incubated with a TAG mixture (triolein:trilinolein = 1:1). Fatty acids released from the TAG mixture were analysed by GC–MS. The amounts of the isolated membranes were normalized to total protein contents. Values represent mean ± SD of three independent experiments, with 5 seeds per experiment. Significant difference between WT and transgenic plants determined using Student’s *t*-test is denoted as *(*P* < 0.1) or **(*P* < 0.05).

Considering that the *SDP1* gene encodes TAG lipase^17^, we hypothesised that the substrate specificity of GmSDP1 plays a major role in determining the ratio of oleic acid to linoleic acid in TAGs. To elucidate the difference in the substrate specificity of GmSDP1 between oleic and linoleic acid, TAG lipase activity in oil body membranes was measured using an equimolar mixture of triolein and trilinolein as substrates. In the WT, the ratio of oleic acid to linoleic acid hydrolysed in this experiment was 2.45:1.00. By contrast, ratios of oleic acid to linoleic acid in the transgenic lines *SDP1i_2*, *_15* and *_19* were 1.4:1.0, 1.1:1.0, and 1.6:1.0, respectively, which were significantly lower than that of the WT (Fig. 6). In addition, the amounts of released fatty acids in the transgenic lines *SDP1i_2*, *_15* and *_19* were decreased to 31.2%, 31.1% and 62.4% of that in WT, respectively (Supplementary Fig. S4). These results indicate that GmSDP1 proteins are one of the major TAG lipases in the oil body membrane, and preferentially cleave oleic acid than linoleic acid.

## Discussion

Increasing the seed oil content has been a major focus in plant breeding. To meet this objective, TAG biosynthetic pathways have been extensively studied in plants^10,12,13^. TAG degradation activity is increased during seed development, although large amounts of TAGs are also produced in the same period. Therefore, the significance of TAG degradation during seed development has been discussed previously^30,31^. Recently, the TAG lipase SDP1 was identified in the oil body membrane^17^, and the suppression of *SDP1* gene expression has been shown to increase the oil content of seeds^25,26^. These reports clearly show that the inhibition of TAG degradation during seed development increases the seed oil content. Moreover, these results indicate that the TAG degradation pathway is activated during seed development, resulting in the degradation of considerable amounts of TAGs. However, the biological significance of TAG degradation in developing seeds remains unclear. In this study, we aimed to understand the significance of TAG degradation during seed development in soybean via RNAi-mediated knockdown of *GmSDP1* expression.

**Figure 7:**
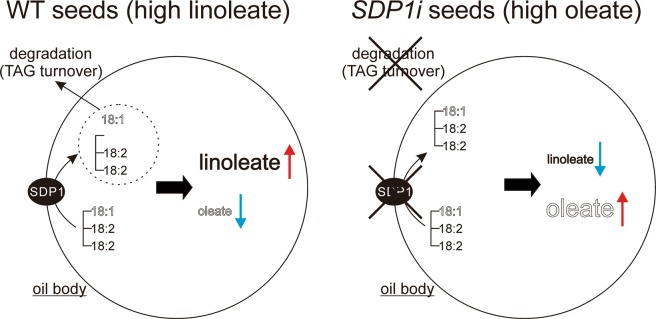
Schematic representation of GmSDP1 function in soybean seeds.

Transgenic soybean seeds were enlarged, and their oil content was higher than that of the WT (Fig. 4A, B, E). Coincidentally, the protein content of the transgenic seeds was smaller than that of WT (Supplementary Fig. S5) This result confirms that the inhibition of TAG degradation increases seed oil content in soybean, which is major oilseed crop in the world. In addition to the increase in seed oil content in transgenic lines, concentrations of oleic and linoleic acids were significantly altered between the transgenic lines and WT (Fig. 5). The concentration of oleic acid in line *SDP1i_15* was approximately 1.6-fold higher than that of the WT, whereas that of linoleic acid was lower for the increment of oleic acid (Fig. 5). Because *GmSDP1* encodes TAG lipase, this result suggests that GmSDP1 preferentially cleaves oleic acid than linoleic acid. This substrate specificity of GmSDP1 was experimentally verified in this study (Fig. 6). Thus, we propose that the cleavage of oleic acid contributes to the low oleic and high linoleic acid content of WT soybean seeds. The fatty acid composition of seeds varies widely among plant species^2^, suggesting that enzymes in the TAG biosynthetic pathway, particularly fatty acid desaturases and elongases, play a key role in the diversity of TAGs^1,2,4,7,9^. By contrast, evidence for the role of the TAG degradation pathway in the fatty acid composition of seeds is lacking. Our results indicate that the substrate specificity of GmSDP1 contributes towards a biased fatty acid composition (low oleic and high linoleic acid) of soybean seeds (Fig. 7). This demonstrates that the TAG degradation pathway, in addition to the TAG biosynthetic pathway, regulates the fatty acid composition of seeds. Thus, the control of fatty acid composition is a major biological significance of TAG degradation during seed development. Coordination between the biosynthesis and degradation of particular fatty acid species leads to a biased and species-specific composition of fatty acids in plant seeds.

Soybean is one of the major oilseed crops; soybean seed oil accounts for one-fourth of the total annual production of plant oil (FAOSTAT: http://faostat3.fao.org/home/). Thus, increasing the oil content of soybean seeds is directly linked to the enhancement of plant oil production. This study showed that the knockdown of *GmSDP1* genes increased the oil yield in soybean (Fig. 4A–F). This increment is consistent with the results in oilseed rape^25^ and jatropha^26^ and confirms that the inhibition of TAG degradation during seed development is one of the effective strategies for producing crop cultivars with high oil content. Additionally, soybean oil is used as cooking oil; thus its fatty acid composition, along with its content, is of commercial importance. Soybean oil contains relatively higher amounts of PUFAs, such as linoleic and linolenic acids, which are prone to oxidation. It is therefore important to decrease the PUFA content of soybean oil to improve its oxidative stability^27^. Deletion of genes encoding fatty acid desaturases has been used as a breeding strategy for reducing the PUFA content of soybean seed oil^27^. This strategy is designed to depress oleic acid desaturation during grain filling^32–34^. In contrast, the knockdown of *GmSDP1* genes is aimed to control oleic acid degradation. Therefore, these two strategies could be compatible; introduction of *SDP1* knockdown may further increase oleic acid content in the conventional high-oleate soybean. Moreover, the knockdown could improve seed oil content without reducing the seed yield.

This shows that genetic modification of *GmSDP1* genes is a novel strategy for generating soybean cultivars with superior oil quantity and quality.

## Methods

### Plant material and growth conditions

The Japanese soybean variety Kariyutaka was obtained from the Hokkaido Prefectural Tokachi Agricultural Experiment Station, Japan and is identical to the resource JP 86520 available from Genebank, National Institute of Agrobiological Science, Japan. Kariyutaka was used as the WT. For germination, seeds were placed on wet paper towel and incubated for 72 h at 22 °C in the dark. Three-day-old seedlings were transferred to 1/5,000a Wagner pots filled with cultured soil and grown under long day conditions (16 h light/8 h dark) at 25 °C in the growth chamber (NK systems, Japan). One-tenth strength of Hoagland solution (1 L) was supplied to the plants once every 3 days in the first month; thereafter, 2 L of the solution was supplied once every 2 days.

### Generation of transgenic plants

The *S1* and *S2* nucleotide sequences were amplified by PCR using sequence-specific primer sets (Supplementary Table S1) and fused using the fusion PCR method. The fused *S1S2* sequence was cloned into the Gateway entry vector pDONR221 (ThermoFisher Scientific, USA) and then transferred to the binary vector pMDC123-GFP^35^. The cauliflower mosaic virus *35S* promoter, *green fluorescent protein* (*sGFP*) gene and *nopaline synthase* (*nos*) terminator in the binary vector were replaced with the soybean *11S globulin* gene promoter.

Recombinant plasmids were infiltrated into WT soybean via *Agrobacterium*-mediated transformation, as described by Yamada *et al*.^35,36^. The primary transformants were designated as T_0_. Transformed soybean lines were selected for Basta resistance and designated as T_1_. The T_2_ progeny exhibiting Basta resistance was used in this study.

### Quantitative real-time PCR (qRT-PCR)

To examine gene expression, qRT-PCR analysis was performed as described previously^37^, with slight modifications. Total RNA was extracted from three independent samples of each transgenic line, according to the method of Kanai *et al*.^38^. Subsequently, cDNA was synthesized from 1 μg of total RNA using the PrimeScript RT Reagent Kit (Takara Bio., Japan) and used for qRT-PCR analysis with KAPA SYBR FAST Universal Kit (KAPA BIOSYSTEMS, USA) and ABI 7500 Fast Real-Time PCR system (ThermoFisher Scientific,), according to the manufacturer’s instructions. Primer sets used for qRT-PCR are listed in Supplementary Table 1.

### Analysis of TAGs and free fatty acids

The analysis of seed TAGs and free fatty acids was performed as described previously^37,39^, with slight modifications. A total of 20 soybean seeds were crushed using a wooden hammer and ground with mortar and pestle. Approximately 10 mg of powdered soybean seeds was ground in 400 μl of chloroform:methanol (2:1) mixture using a stainless steel homogenizer. The resulting extract was centrifuged at 2,000 × *g* for 5 min. The supernatant was collected, and the pellet was extracted with 400 μl of chloroform:methanol (2:1) mixture by vortexing. The centrifugation step was repeated, and the supernatant was combined with the first supernatant, dried and stored at –20 °C. Dried lipids were dissolved in 25 μl hexane and spotted onto a silica gel thin-layer chromatography (TLC) plate (Merck Millipore; http://www.merckmillipore.com) to separate TAGs and free fatty acids from total lipids. Triheptadecanoin (Wako, Japan) and oleic acid (Sigma-Aldrich, USA) was used as standards. TLC separation was carried out using hexane:diethylether:acetic acid (80:20:1) mixture. TAG and free fatty acid spots were collected and extracted using 100 μl hexane. Fatty acid methyl esterification was performed using a Fatty Acid Methylation Kit (Nacalai Tesque, Japan). Fatty acid methyl esters were quantified via gas chromatography (GC-2010, Shimadzu, Japan)–mass spectrometry (JMSDX-300, JEOL, Japan) using a DB-23 column (Agilent Technology, USA).

### Enzyme assay

Oil body membrane was purified from developing seeds of the WT and transgenic lines 35 DAF, as described previously by Edmond^40^. Protein content of the oil body fraction was measured using the Bio-Rad Protein Assay (Bio-Rad, USA). Lipase activity was determined on the basis of the amount of fatty acids produced after TAG hydrolysis. Reaction buffer (400 μl) containing 50 mM Bis-Tris propane (pH 8.1), 2 mM DTT, 1 mM CaCl_2_, 5 mM triolein (Sigma-Aldrich) and 5 mM trilinolein (Sigma-Aldrich, USA). Substrates were emulsified with 5% (w/v) gum arabic (Wako, Japan), followed by ultrasonic treatment for 30 s at 20 W using Sonifier (Branson, USA). Reactions were carried out on a shaking incubator for 60 min at 30 °C and 120 rpm.

## Supplementary information

Supplementary information
