# Supplementary material for: Soybean (Glycine max L.) triacylglycerol lipase GmSDP1 regulates the quality and quantity of seed oil
Source: Sci Rep. 2019 Jun 20;9:8924. doi: 10.1038/s41598-019-45331-8 (PMC6586785; doi:10.1038/s41598-019-45331-8)
Supplement: Supplementary file 1 — Supplementary information [file 41598_2019_45331_MOESM1_ESM.pdf]

Soybean (*Glycine max* L.) triacylglycerol lipase GmSDP1 regulates the quality and quantity of seed oil

Masatake Kanai[1], Tetsuya Yamada[2], Makoto Hayashi[3], Shoji Mano[1,4*] and Mikio Nishimura[5*]

[1] Laboratory of Organelle Regulation, National Institute for Basic Biology, Okazaki 444-8585, Japan

[2] Graduate School of Agriculture, Hokkaido University, Sapporo 060-8589, Japan

[3] Department of Bioscience, Nagahama Institute of Bio-Science and Technology, Nagahama 526-0829, Japan

[4] Department of Basic Biology, SOKENDAI (The Graduate University for Advanced Studies), Okazaki 444-8585, Japan

[5] Department of Biology, Faculty of Science and Engineering, Konan University, Kobe 658-8501, Japan

Primer name	Sequence
For cloning	
B1N-GmSDP1-1_CDS_F	AAAAAGCAGGCTTCATGGATCATATTAGTAATGAGGCTAGTA
B2N-GmSDP1-1_CDS_R	AGAAAGCTGGGTCCTAACTGTCAACAATGCTCTGA
B1N-GmSDP1-2_CDS_F	AAAAAGCAGGCTTCATGATGGATCATATTAGTAATGAGG
B2N-GmSDP1-2_CDS_R	AGAAAGCTGGGTCCTAACTGTCAACAATGCTTTGATC
B1N-GmSDP1-3_CDS_F	AAAAAGCAGGCTTCATGGATCATATAAGTAACGAGGCTAG
B2N-GmSDP1-3_CDS_R	AGAAAGCTGGGTCTTAGGAAGCTGAACTAGCAGCATC
B1N-GmSDP1-4_CDS_F	AAAAAGCAGGCTTCATGGATCATATAAATAACGAGGCTAG
B2N-GmSDP1-4_CDS_R	AGAAAGCTGGGTCTTAATTGTCAGCAATGCTTTGATC
B1N-GmEF1b_CDS_F	AAAAAGCAGGCTTCATGGCTGTCACCTTCTCAGATC
B2N-GmEF1b_CDS_R	AGAAAGCTGGGTCTTAAATTTTGTTGAATGCAACAATG
For quantitative RT–PCR	
GmSDP1-1_Q_F	AGACGTTGATACCGATCAAAAC
GmSDP1-1_Q_R	GGCTGCAAAAGATCACCTTC
GmSDP1-2_Q_F	ATCGGCAGTTCCAGCAGAG
GmSDP1-2_Q_R	GCCTGGGGCTATGATACTGA
GmSDP1-3_Q_F	GAAAATGGCAACAGGATTGTC
GmSDP1-3_Q_R	TAGCAGCATCCACCTCCTTC
GmSDP1-4_Q_F	ATGCTGCTGCTGCTGCTAC
GmSDP1-4_Q_R	CATCAAAGGCCCACCAGAT
GmEF1b_Q_F	ACGCTCAAGGGGTAAGATTC
GmEF1b_Q_R	TCCTCTGTCTCATCACCAAAG

Supplementary Table S1. Nucleotide sequences for primers used in this study.

Supplementary Figure S1. The Alignment of amino acid sequences between AtSDP1 and four GmSDP1s.

The alignment was performed using ClustalW (version 2.1). Asterisk denotes conserved residue in all sequences in the alignment.

```
AtSDP1          -MD-ISNEASVDPFSIGPSSIMGRTIAFRVLFCRSMSQLRRDLFRFLLHWFLRFKLTVSP 58
GmSDP1-1        -MDHISNEASVDPFAIGPSSILGRTIAFRVLFCKSISQLRYHILFVLSNIFKRFRGFWAP 59
GmSDP1-2        -MDHINNEASVDPFAIGPSSILGRTIAFRVLFCKSISQLRHHIHFVLLDILKRFWGFWGP 59
GmSDP1-3        -MDHISNEASIDRFPIGPSDILGRTIAFRVLFCKSISHFRHHIFLVLLDLFYRFRGGLAS 59
GmSDP1-4        MMDHISNEASVDRFPIGPSGILGRTIAFRVLFCKSISHFRHHIFIVLLDLFYRFRGGLAS 60
                 ** * **** * * **** * *********** * *  *      *     **

AtSDP1          FVSWFHPRNPQGILAVVTIIAFVLKRYTNVKIKAEMAYRRKFWRNMMRTALTYEEWAHAA 118
GmSDP1-1        IVSWLHPRN-QGILAMMTMIAFLLKRYTSVKVRANIAYKRKFWRNMVRAALTYEEWAHAA 118
GmSDP1-2        IVSWLHPRNPQGILAMMTIIAVLLKRYSSVKVRAEIAYRQNFWRNMMRAALTYEEWAHAA 119
GmSDP1-3        FISWLHPRNPQGILAMMTIVAFLLKRYTNVKARAEMAYRRKFWRNMMRSALTYDEWAHAA 119
GmSDP1-4        FISWLHPRNPQGILAMMTIVAFLLKRYTNVKSRAEMAYRRKFWRNMMRSALTYEEWAHAA 120
                  ** **** *****  *  *   ***  **  *  **   ***** * **** ******

AtSDP1          KMLEKETPKMNES-DLYDEELVKNKLQELRHRRQEGSLRDIMFCMRADLVRNLGNMCNSE 177
GmSDP1-1        KMLDRETQKMNES-DLYDVELVSNKLEELRHRRQEGSLRDIIFCMRADLIRNLGNMCNPE 177
GmSDP1-2        KMIDRETPKMNESKDLYDVELVSNKLQELRHRRQEGSLRDIIFCMRADLVRNLGNMCNPE 179
GmSDP1-3        KMLDKETTKMNES-DLYDVELVRNKLQELRHRRQEGSLRDIMFCMRADLIRNLGNMCNPE 178
GmSDP1-4        KMLDKETTKMNES-DLYDVELVRNKLQELRHRRQEGSLGDIMFFMRADLIRNLGNMCNPE 179
                **   ** ***** **** *** *** *********** ** * ***** ******** *

AtSDP1          LHKGRLQVPRHIKEYIDEVSTQLRMVCNSDSEELSLEEKLSFMHETRHAFGRTALLLSGG 237
GmSDP1-1        LHKARLQMPRLIKEYIDEVSTQLRMVCNSDSEELALEEKLSFMHETRHAFGRTALLLSGG 237
GmSDP1-2        LHKGRLQMPRLIKEYIDEVSTQLRMVCNSDSEELALEEKLSFMHETRHAFGRTALLLSGG 239
GmSDP1-3        LHKGRLQVPKLIKEYIDEVTTQLRMVCDSDSEELSLEEKLAFMHETRHAFGRTALLLSGG 238
GmSDP1-4        LHKGRLQVPKLIKEYIDEVTTQLRMVCDSDSEELSLEEKLAFMHETRHAFGRTALLLSGG 239
                *** *** *  ******** ******* ****** ***** *******************

AtSDP1          ASLGAFHVGVVRTLVEHKLLPRIIAGSSVGSIICAVVASRSWPELQSFFENSLHSLQFFD 297
GmSDP1-1        ASLGAFHVGVVKTLVEHKLLPRIIAGSSVGSIMCSIVATRSWPELQSFFEDSLHSLQFFD 297
GmSDP1-2        ASLGAFHVGVVKTLVEHKLLPRIIAGSSVGSIMCSIVATRSWPELQSFFEDSLHSLQFFD 299
GmSDP1-3        ASLGASHVGVVKTMVEHKLMPRIIAGSSVGSIMCAVVATRTWPELQSFFEDSWHSLQFFD 298
GmSDP1-4        ASLGASHVGVVKTLVEHKLMPRIIAGSSVGSIMCAVVATRTWPELQSFFEDSWHSLQFFD 299
                ***** ***** * ***** ************ *  ** * *********** *******

AtSDP1          QLGGVFSIVKRVMTQGALHDIRQLQCMLRNLTSNLTFQEAYDMTGRILGITVCSPRKHEP 357
GmSDP1-1        QMGGIFTVVKRVTTYGAVHEIRQLQMLLRHLTSNLTFQEAYDMTGRILGITVCSPRKHEP 357
GmSDP1-2        QMGGIFTVVKRVTTYGAVHEIRQLQMLLRNLTSNLTFQEAYDMTGRILGITVCSPRKHEP 359
GmSDP1-3        QMGGIFAVVKRVTTLGAVHEIRQLQMMLRHLTSNLTFQEAYDMTGRILGITVCSPRKHEP 358
GmSDP1-4        QMGGIFAVVKRVTTLGAVHEIRQLQMMLRHLTSNLTFQEAYDMTGRILGITVCSPRKHEP 359
                * ** *  **** * ** * *****  *********************************

AtSDP1          PRCLNYLTSPHVVIWSAVTASCAFPGLFEAQELMAKDRSGEIVPYHPPFNLDPEVGTKSS 417
GmSDP1-1        PRCLNYLTSPHVVIWSAVTASCAFPGLFEAQELMAKDRSGEIVPYHPPFNLGPEKG--ST 415
GmSDP1-2        PRCLNYLTSPHVVIWSAVTASCAFPGLFEAQELMAKDRSGEIVPYHPPFNLGPEKG--ST 417
GmSDP1-3        PRCLNYLTSPHVVIWSAVTASCAFPGLFEAQELMAKDRSGEIIPYHPPFNLGPEEG--ST 416
GmSDP1-4        PRCLNYLTSPHVVIWSAVTASCAFPGLFEAQELMAKDRSGEIVPYHPPFNLGPEEG--ST 417
                ****************************************** ******** ** *  *

AtSDP1          SGRRWRDGSLEVDLPMMQLKELFNVNHFIVSQANPHIAPLLRLKDLVRAYGGRFAAKLAH 477
GmSDP1-1        SVRRWRDGSLENDLPMMQLKELFNVNHFIVSQANPHIAPLLRLKEFVRAYGGNFAAKLAH 475
GmSDP1-2        SVRRWRDGSLEMDLPMMQLKELFNVNHFIVSQANPHIAPLLRFKEIIRAYGGNFAAKLAH 477
GmSDP1-3        PARRWRDGSLEIDLPMMQLKELFNVNHFIVSQANPHIAPLLRLKEFVRTYGGNFAAKLAH 476
GmSDP1-4        PVRRWRDGSLEIDLPMMQLKELFNVNHFIVSQANPHIAPLLRLKEFVRTYGGNFAAKLAH 477
                  ********* ****************************** *   * *** *******

AtSDP1          LVEMEVKHRCNQVLELGFPLGGLAKLFAQEWEGDVTVVMPATLAQYSKIIQNPTHVELQK 537
GmSDP1-1        LAEMEVKHRCNQVLELGFPLGGLAKLFAQDWEGDVTVVMPATLAQYLKIIQNPSYVELQK 535
GmSDP1-2        LAEMEVKHRCNQVLELGFPLGGLAKLFAQDWEGDVTVVMPATPAQYLKIIQNPSYVELQK 537
GmSDP1-3        LVEMEVKHRCNQILELGFPLGGLAKLFAQDWEGDVTVVIPATLAQYTKIIQNPSYGELQK 536
GmSDP1-4        LVEMEVKHRCHQILELGFPLGGLAKLFAQDWEGDVTVVIPATLAQYTKIIQNPSYGELQK 537
                * ******** * **************** ******** ******* ******   ****

AtSDP1          AANQGRRCTWEKLSAIKSNCGIELALDDSVAILNHMRRLKKSAERAATATSSSHHGLAST 597
GmSDP1-1        AANQGRRCTWEKLSAIKANCGIELALDESVAILNHMRRLKRNAERVATAS----HGLPST 591
GmSDP1-2        AANQGRRCTWEKLSAMKANCGIELALDESVAILNHMRRLKRNAERTAAAS----HGLPST 593
GmSDP1-3        ATNQGRRCTWEKLSAIKANCGIELALDECVVILNHMRRLKRIAERAASAS----QGLSST 592
GmSDP1-4        AANQGRRCTWEKLSAIKANCGIELALDECVVILNHMRRLKRIAERAASAS----HGLSST 593
                * ************* * *********  * *********  *** * *      ** **

AtSDP1          TRFNASRRIPSWNVLARENSTGSLDDLVTDNNL--------HASSGRN----------- 637
GmSDP1-1        VKFSGSRRIPSWNIIARENSTGSLEDLHADAAS--SLHQGVISPSGATGKNWKSHRIIHD 649
GmSDP1-2        GKFSGSRRIPSWNIIARENSTGSLEDLHAAAAATSSLHQGVISPSGATGKNWKSHRSFHD 653
GmSDP1-3        VRFSASKRIPSWNCIARENSTGSLEDLTDVAAS---KHQGISSSCGTNGKTWKTYRGILD 649
GmSDP1-4        VRFSASKRIPSWNCIARENSTGSLEDLTDVASS---LHQGIGSSSRANGKTWKTHRGIHD 650
                  *  * ******  ********* **

AtSDP1          LSDSETESV-ELSSWTRTGGPLMRTASANKFIDFVQSLD---IDIALV-RGFSSS-PNSP 691
GmSDP1-1        ASDSESETA-DLNSWTRSGGPLMRTTSADMFIDFVQDLEFHKVNTELN-RGKVT--HTSP 705
GmSDP1-2        ASDSESESAHDLNSWTRSGGPLMRTTSADMFIDFFQNIE---VDTELN-RGKVTNTTTSP 709
GmSDP1-3        GSDSDSESV-DLHSWTRSGGPLMRTTSANMFVDFLQNLD---VDTDQNNKGLVSR--ANP 703
GmSDP1-4        GSDSDSESV-DLHSWTRTGGPLMRTTSANMFVDFLQNLE---VDTDPN-KGLVSH--TIH 703
                 ***  *    * **** ******* ** ** ** *              *

AtSDP1          AVPPGGSFTPSPRSIAAHS----DIESNSNSNN-LGTSTSSITVTEGDLLQPERTSNGFV 746
GmSDP1-1        HDFQHHSSRHTT--VNRWS-ESTENDQKENGNR-IVMNESNIMVTEGDLLQPERIHNGIV 761
GmSDP1-2        RDFQHHISRHTTPDHNRWCSKRTEIDQKENGNRMIAMNGSNIMVTEGDLLQPERIHNGIV 769
GmSDP1-3        NDFQYRSPRLATLDRNSDS---TESEPREIGNR--VFNVSSILVTEGDLLQPERIHNGIV 758
GmSDP1-4        NDFQYHSPRLTTLDRNSDS---TESEPRETGNR--VVNVSSILVTEGDLLQPERIHNGIV 758
                                               *         * ***********  ** *

AtSDP1          LNVVKR------ENLGMPSIGNQNTELPESVQLDIPEKEMDCSSVSEHEEDDNDNEEEHN 800
GmSDP1-1        FNVVKK-ENLTP-NRNHDYN-SYNNEVAECVQIDFPGKEVDAASSAS------------- 805
GmSDP1-2        FNVVKKQDFLTPSNRNNDYNSCCNNEVAECVQIEFPGKEVDALSSASENGDDG-STMARS 828
GmSDP1-3        FNVVKK-EDLSPLSGSSHDFENYNIEVAECVQDECPGKEIDAASSASEHGDDEESTVARS 817
GmSDP1-4        FNVVKK-EDLSPLSSSSHGFENYNIEVAECVQDECPGKEIDAASSASEHGDDEESMPARS 817
                 ****                  * *  * **   * ** *  *

AtSDP1          GSSLVTVSS--EDSGLQEPVSGSVIDA 825
GmSDP1-1        ---------------------------
GmSDP1-2        LTEKPDYNSTNHFS-KDFGEDQSIADN 854
GmSDP1-3        LTETQDYNSMDHHS----GMDQSIVDS 840
GmSDP1-4        LTDMPDYNSIDHHSGTDSGMDQSIVDS 844
```

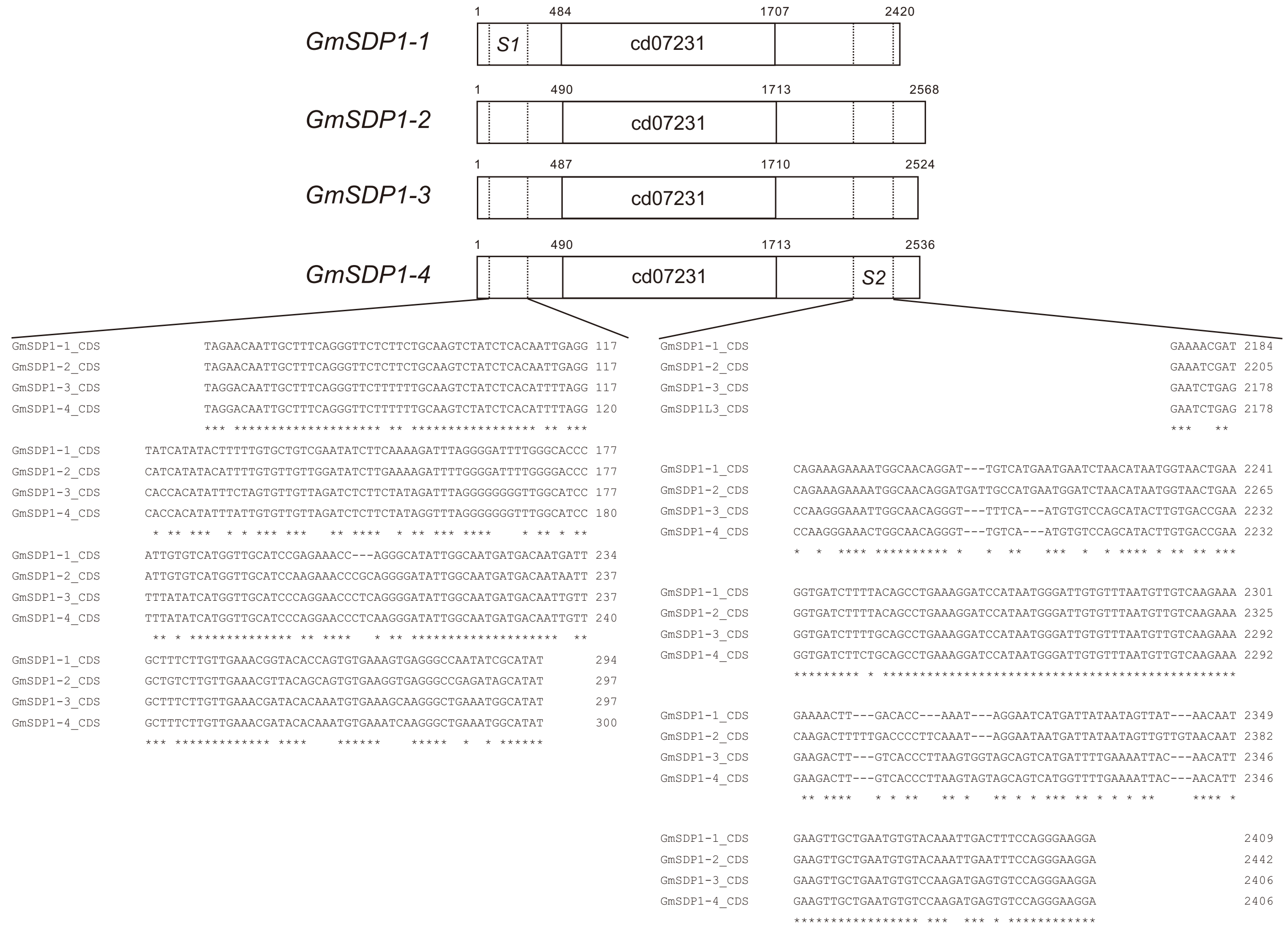

Supplementary Figure S2. Schematic diagram showing the positions of *S1* and *S2* sequences used in the *SDP1* RNAi construct.

The positions and sequence alignments of the *S1* and *S2* loci are shown in the *GmSDP1s* . The nucleotide sequences of *S1* and *S2* were highly conserved among four *GmSDP1* cDNAs. The *S1* sequence used to produce the *SDP1* RNAi construct was cloned from *GmSDP1-1* cDNA, and the *S2* sequence was cloned from *GmSDP1-4* cDNA.

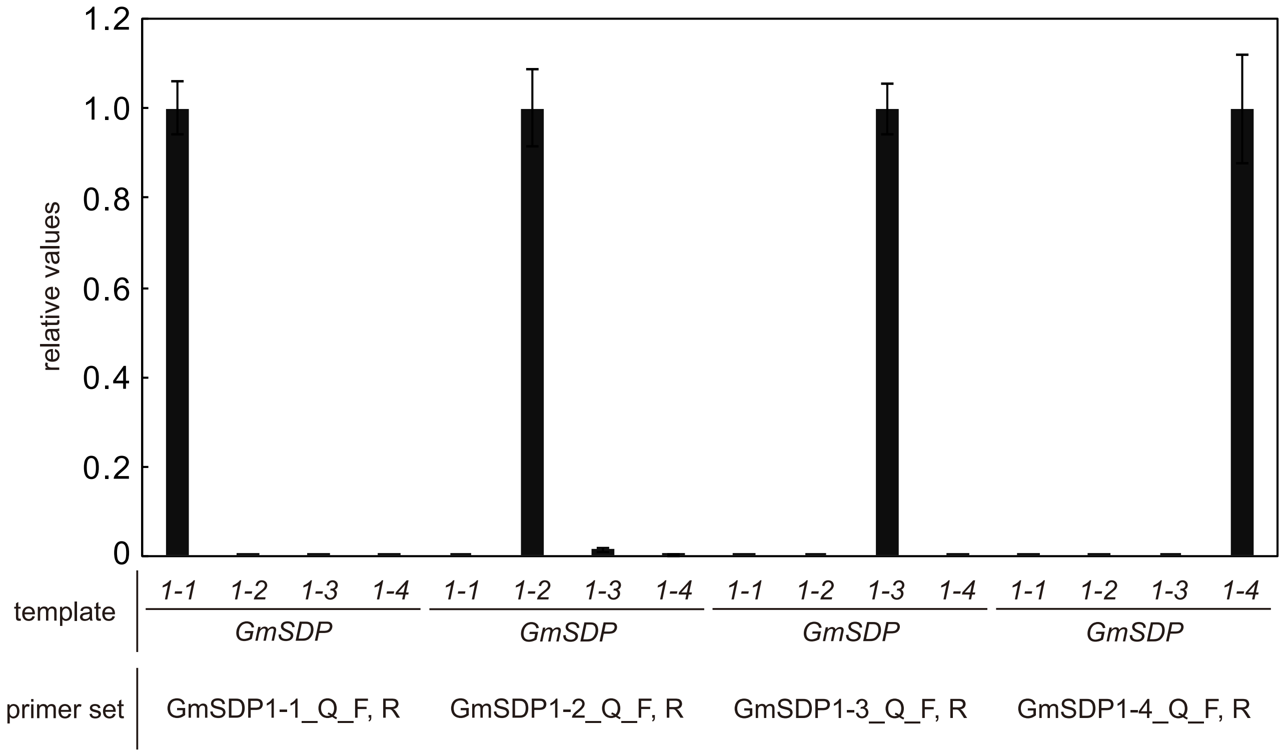

Supplementary Figure S3. Specificity of primers used in quantitative RT-PCR in this study.

Specificity of primer sets for measuring *GmSDP1-1*, *1-2*, *1-3*, and *1-4* transcript levels was tested. This quantitative RT-PCR analysis was performed with each primer set (GmSDP1-1_F and _R, GmSDP1-2_Q_F and _R, GmSDP1-3_Q_F and _R, and GmSDP1-4_Q_F and _R). The DNA solution containing 6×10^5 copies/μl of plasmids with the full-length *GmSDP1-1*, *1-2*, *1-3*, or *1-4* cDNA were prepared and used as templates. Values represent mean ± SD of three individual measurements.

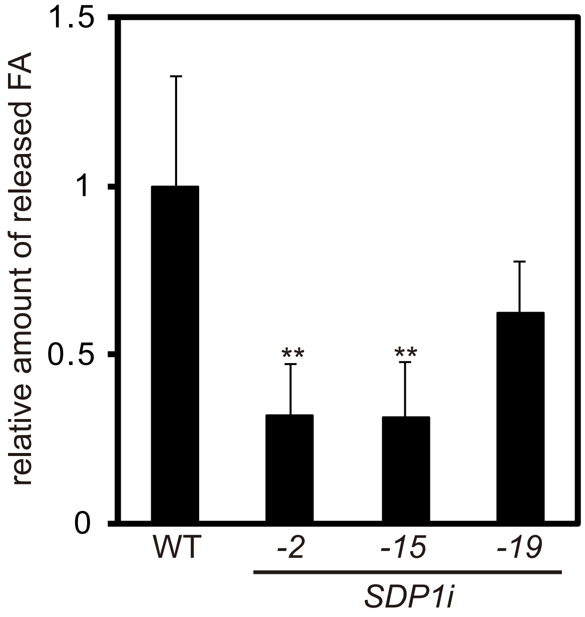

Supplementary Figure S4. Relative values of lipase activity in oil body membranes in soybean.

Relative activity of TAG lipase was determined using oil body membranes isolated from seeds at 35 DAF. The isolated membranes were incubated with a TAG mixture (triolein:trilinolein = 1:1). Total amounts of fatty acids released from the TAG mixture were measured by GC–MS. The amounts of the isolated membranes were normalized to total protein contents.

Values represent mean ± SD of three independent experiments, with 5 seeds per experiment. Significant difference between WT and transgenic plants determined using Student's t-test is denoted as ** ($P < 0.05$).

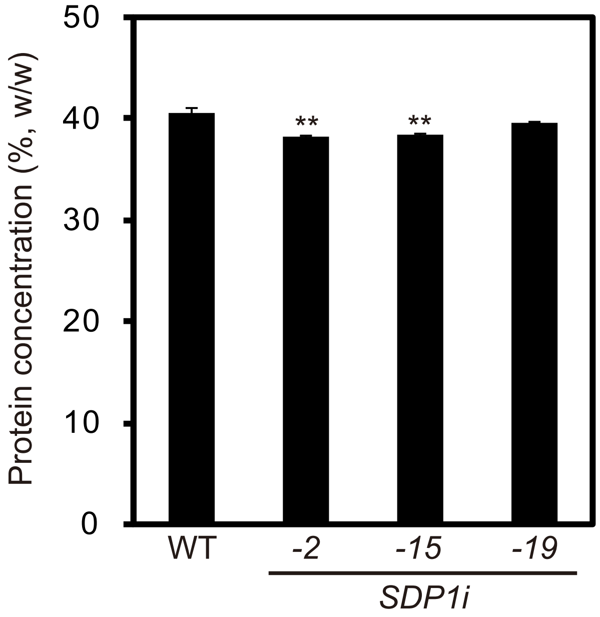

Supplementary Figure S5. Protein concentrations of seeds of transgenic lines

Protein concentrations were measured by BCA protein assay. Values represent mean ± SD of three independent experiments, with 20 seeds per experiment. Significant difference between WT and transgenic plants determined using Student's t-test is denoted as ** ($P < 0.05$).
